# Prognostic value of preoperative mGPS, CAR, and NLR in patients undergoing resection for non-small cell lung cancer

**DOI:** 10.17305/bb.2026.14295

**Published:** 2026-06-18

**Authors:** Mehmet Mermer, Hıdır Esme

**Affiliations:** 1 Department of Chest Diseases, Konya City Hospital, Konya, Türkiye; 2 Department of Thoracic Surgery, Konya City Hospital, Konya, Türkiye

**Keywords:** Non–small cell lung cancer, C-reactive protein to albumin ratio, neutrophil-to-lymphocyte ratio, systemic inflammation, modified Glasgow prognostic score, surgical resection, overall survival, prognostic biomarkers, platelet-to-lymphocyte ratio, platelet-to-albumin ratio

## Abstract

Systemic inflammation is increasingly recognized as a crucial prognostic factor in non-small cell lung cancer (NSCLC). However, comprehensive evaluations comparing multiple inflammation-based indices in surgically treated cohorts, especially across specific time-dependent survival endpoints, remain scarce. This retrospective observational cohort study analyzed 270 NSCLC patients who underwent surgical resection of the primary tumor within the Konya City Hospital healthcare network. Preoperative inflammatory indices calculated included C-reactive protein to albumin ratio (CAR), neutrophil-to-lymphocyte ratio (NLR), platelet-to-lymphocyte ratio (PLR), platelet-to-albumin ratio (PAR), and modified Glasgow Prognostic Score (mGPS). We assessed overall survival and time-specific survival at 1, 2, and 5 years. Group comparisons were conducted based on survival status at each time point, utilizing univariate and multivariable logistic regression to identify factors associated with time-specific survival. The mean age of the cohort was 71.64 ± 9.71 years, with 78.9% being male. Lobectomy was the most frequent procedure (72.6%). During follow-up, 145 patients died (overall mortality rate of 53.7%). Among these, the mean time from surgery to death was 32.63 ± 23.83 months. Survival rates at 1, 2, and 5 years were 90.0%, 79.3%, and 53.3%, respectively. Inflammatory indices were consistently elevated in non-survivors across all evaluated time points. In multivariable models, CAR and high mGPS status were independently associated with reduced 1-year survival. At 2 years, CAR and high mGPS status maintained their independent association with diminished survival, while NLR demonstrated borderline significance. For 5-year survival, high mGPS status and NLR remained independently associated with reduced survival. Unadjusted analyses revealed a graded association between mGPS and survival. Our findings indicate that preoperative systemic inflammation, particularly elevated mGPS status, CAR, and NLR, is associated with mortality at multiple postoperative time points following NSCLC resection. These results underscore the potential prognostic utility of readily available inflammation-based biomarkers and necessitate further validation through prospective investigations.

## Introduction

Non–small cell lung cancer (NSCLC) remains the leading cause of cancer-related mortality globally. Despite advancements in systemic and surgical treatments, long-term survival for many patients with NSCLC remains limited [1]. Traditional prognostic factors, such as tumor stage, histopathology, and lymph node status, inadequately account for the observed heterogeneity in outcomes among surgically treated patients. Recent evidence increasingly highlights the crucial role of systemic inflammation in lung cancer progression and survival.

Inflammation-based biomarkers, derived from routine laboratory parameters, have emerged as promising prognostic tools. Among these, the C-reactive protein to albumin ratio (CAR) has garnered significant attention as an integrated marker reflecting both systemic inflammatory response and nutritional status. A meta-analysis demonstrated a significant association between elevated CAR and poorer overall survival in lung cancer patients [2]. Similarly, retrospective analyses in operable NSCLC have indicated that an increased preoperative CAR independently predicts long-term disease progression and mortality [3]. In elderly patients undergoing surgical resection, preoperative CAR has also been identified as an independent prognostic factor for overall survival [4]. Furthermore, prospective cohort data suggest that post-diagnostic C-reactive protein, albumin, and their ratio strongly predict mortality in NSCLC patients [5].

Other inflammation-based indices, including the neutrophil-to-lymphocyte ratio (NLR) and platelet-to-lymphocyte ratio (PLR), have similarly demonstrated prognostic significance. An elevated preoperative NLR has been shown to independently predict reduced survival following complete resection for NSCLC [6]. In primary operable disease, NLR has been validated as an independent predictor of overall survival and recurrence-free survival [7]. A systematic review and meta-analysis further confirmed that an increased PLR is associated with shorter overall survival in NSCLC [8]. Moreover, composite indices, such as the systemic immune-inflammation index, have also demonstrated independent prognostic value in surgically resected NSCLC [9].

The Glasgow Prognostic Score (GPS) and its modified version (mGPS), which integrate C-reactive protein and albumin into a categorical inflammation-based score, have been extensively investigated in lung cancer. GPS has been shown to independently predict overall survival in patients with resectable NSCLC [10]. A comprehensive meta-analysis further supported the prognostic relevance of mGPS across lung cancer populations, although the magnitude of effect appeared to vary with treatment modality [11]. Collectively, these findings suggest that systemic inflammatory status can provide clinically meaningful prognostic information beyond traditional tumor-related factors.

Despite growing evidence supporting the prognostic role of inflammation-based indices, comparative evaluation of multiple inflammatory markers within a single surgically treated cohort and assessment of their impact on short-, intermediate-, and long-term survival remain limited. Most studies have focused on overall survival without specifically examining predefined time-dependent mortality endpoints such as 1-, 2-, and 5-year survival.

Therefore, the present study aimed to evaluate the association between preoperative systemic inflammatory markers, including CAR, NLR, PLR, platelet-to-albumin ratio (PAR), and mGPS, and time-specific survival outcomes at 1, 2, and 5 years in patients undergoing surgical resection for NSCLC. By comprehensively analyzing these markers within a well-defined surgical cohort, we sought to evaluate their relative associations with early and long-term mortality.

## Materials and methods

### Study design and setting

This retrospective observational cohort study was conducted at Konya City Hospital. The study evaluated patients diagnosed with NSCLC between 2010 and 2018 who underwent surgical resection of the primary tumor. Clinical, laboratory, histopathological, and survival data were retrieved from the hospital information system and laboratory information system using a predefined standardized data extraction template. Extracted variables were verified against original source records to ensure accuracy and consistency. All data were stored on password-protected institutional systems in accordance with data security regulations.

The study cohort was originally treated at Konya Education and Research Hospital between 2010 and 2018. Following the relocation of major clinical services from Konya Education and Research Hospital to Konya City Hospital in 2020, historical patient records were transferred to and incorporated into the institutional electronic archive systems accessible through Konya City Hospital. Therefore, the study database was derived from institutional archival records originating from the predecessor hospital and subsequently maintained within the Konya City Hospital information infrastructure. All clinical, laboratory, surgical, histopathological, and follow-up records included in the analysis originated from patients who received diagnosis, treatment, and follow-up within the same institutional healthcare network. No external research registries, third-party databases, or independently maintained repositories were used for patient identification or data extraction. Data retrieval was performed directly from the institutional hospital information system and associated laboratory information system archives.

### Study population

Hospital records identified 491 patients diagnosed with NSCLC during the study period. Among these, 270 patients who underwent surgical resection of the primary lung tumor (pneumonectomy, bilobectomy, lobectomy, or wedge resection) and had complete baseline laboratory and survival data met eligibility criteria and were included in the final analysis (Figure 1). Patients were followed from the date of surgery until death or last documented follow-up. All surgical procedures were performed with curative therapeutic intent according to the multidisciplinary treatment strategy documented at the time of surgery. Surgical management included standard oncological resections, and lymph node sampling and/or mediastinal lymph node dissection was performed according to institutional thoracic surgery practices and prevailing clinical guidelines during the study period.

**Figure 1.**
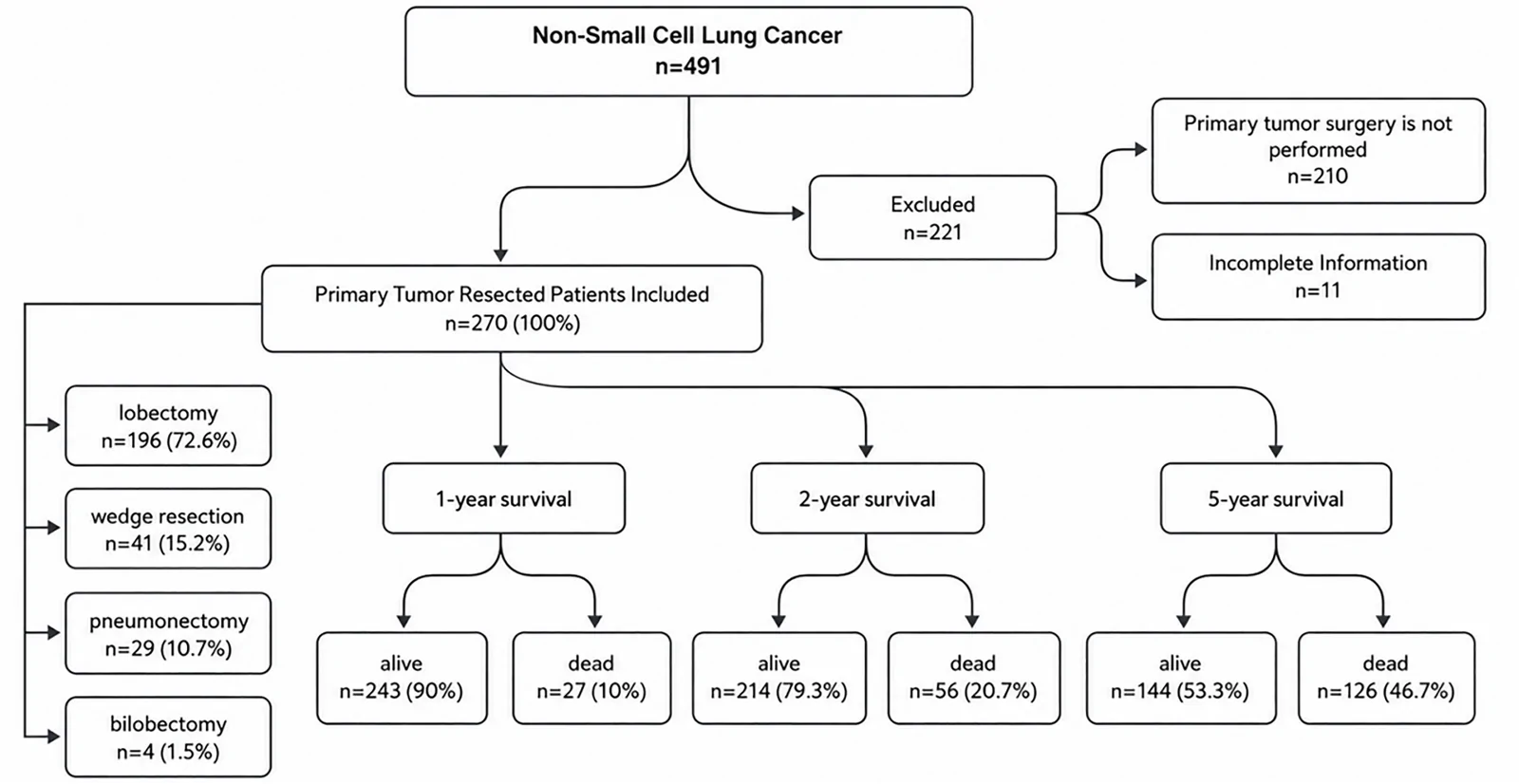
**Study flow diagram.** This flowchart illustrates the patient selection and inclusion process. Out of 491 patients initially diagnosed with non-small cell lung cancer, 270 who underwent primary tumor resection and met all eligibility criteria were included in the final analysis. Excluded patients comprised those who did not undergo surgery (*n* ═ 210) and those with incomplete data (*n* ═ 11). The diagram details surgical procedures and presents 1-, 2-, and 5-year survival outcomes.

The small number of stage IVA and IVB patients included in the cohort represented highly selected surgical candidates who underwent resection following multidisciplinary evaluation and individualized treatment planning. Surgical treatment in these cases was performed according to contemporary clinical decision-making practices, including selected advanced-stage presentations considered potentially amenable to surgical management.

Eligible cases were included consecutively to minimize selection bias. In patients with more than one hospitalization, only the index surgical admission was considered for analysis to avoid duplicate observations.

### Eligibility criteria

Patients were eligible if they had histologically confirmed NSCLC, documented clinical stage at presentation, surgical treatment of the primary tumor, available baseline laboratory values (including platelet count, neutrophil count, lymphocyte count, C-reactive protein, and albumin), and available survival status data. Patients who did not undergo surgery, lacked essential laboratory data, or did not have documented survival information were excluded. Additionally, patients with active infection, those who received preoperative blood transfusion, and those using medications that could influence inflammatory status, such as corticosteroids, were also excluded. No imputation procedures were applied.

### Definition of exposures and covariates

Baseline inflammatory parameters were obtained from the last available preoperative laboratory assessment performed within 30 days before surgery. When multiple laboratory measurements were available during this interval, the value closest to the date of surgery was used. Laboratory parameters were obtained before surgical intervention and reflected routine preoperative clinical evaluation. The mGPS was calculated using serum C-reactive protein (CRP) and albumin levels. Patients with CRP ≤10 mg/L were assigned an mGPS of 0, regardless of albumin concentration. Patients with CRP >10 mg/L and albumin ≥3.5 g/dL were assigned an mGPS of 1. Patients with CRP >10 mg/L and albumin <3.5 g/dL were assigned an mGPS of 2. These categories were mutually exclusive and collectively exhaustive. The mGPS was analyzed as a categorical variable. For regression analyses, mGPS was additionally dichotomized as low mGPS (score 0) and high mGPS (scores 1–2) to improve interpretability and model stability. The original three-category classification was retained for descriptive analyses and Kaplan–Meier visualization.

Laboratory measurements included CRP (mg/L), albumin (g/dL), neutrophil count (×10^9^/L), lymphocyte count (×10^9^/L), and platelet count (×10^9^/L), all obtained from routine preoperative blood testing performed in the institutional clinical laboratory. Additional inflammatory indices were calculated as derived variables. The CAR was calculated by dividing CRP (mg/L) by albumin (g/dL). The NLR was calculated as the absolute neutrophil count divided by the absolute lymphocyte count. The PLR was calculated as the absolute platelet count divided by the absolute lymphocyte count. The PAR was calculated as the absolute platelet count divided by serum albumin concentration.

Covariates included age, sex, stage group (categorized as early [stage I–II] or advanced stage [stage III–IV]), histopathological subtype, type of surgical procedure, chemotherapy status, and radiotherapy status. Chemotherapy and radiotherapy variables reflected treatment information available within the retrospective database. However, treatment timing was not uniformly documented; therefore, these variables could not be reliably classified as neoadjuvant, adjuvant, postoperative, or other treatment categories. Detailed clinical stage distributions were reported according to tumor-node-metastasis classification (TNM) categories, whereas grouped stage classifications were used in selected analyses to improve statistical stability within smaller subgroups.

### Outcome definitions

The primary outcome was overall survival, defined as the time from surgical resection to death from any cause. Patients alive at the last follow-up were censored on that date. Survival time was calculated in months. The median follow-up duration for the cohort was 73.5 months (range, 1–189 months), with the final follow-up date for survival ascertainment being October 1, 2025.

In addition to overall survival, time-specific survival endpoints were evaluated. One-year, two-year, and five-year survival statuses were determined based on patient survival at 12, 24, and 60 months post-surgery, respectively. For these fixed time points, patients were categorized as alive or deceased based on documented survival status. Survival status at each predefined time point was available for all included patients; no patient was lost to follow-up before the 5-year endpoint, ensuring complete ascertainment of survival status at 1, 2, and 5 years.

### Bias and confounding

Selection bias was minimized by including all consecutive eligible surgical cases during the study period. Information bias was reduced through the use of standardized electronic records and predefined variable definitions. Given the retrospective nature of the study, residual confounding cannot be entirely excluded. Multivariable regression models were applied to adjust for potential confounders.

### Study size

The sample size comprised all consecutive patients meeting eligibility criteria during the study period. As this was a retrospective investigation, no *a priori* sample size calculation was performed.

### Missing data

A complete-case analytical approach was employed. Patients with missing key exposure or outcome variables were excluded from the analysis. No data imputation techniques were implemented.

#### Ethical statement

All procedures performed in this study adhered to the ethical principles of the Declaration of Helsinki and its subsequent amendments. Ethical approval was obtained from the Institutional Review Board of Konya City Hospital (approval number: 2026/25; approval date: February 09, 2026). This approval encompassed the retrospective evaluation of archived clinical, laboratory, surgical, histopathological, and survival records accessible through the institutional electronic medical record systems and archival databases of Konya City Hospital, including historical records transferred from Konya Education and Research Hospital following institutional integration procedures. The ethics committee specifically authorized the use of these retrospective records for research purposes under anonymized data-processing procedures. As the investigation relied exclusively on retrospectively collected and fully de-identified data, the requirement for informed consent was waived. All personal identifiers were removed prior to analysis, and data were processed in an anonymized form.

**Table 1:** Baseline demographic and clinical characteristics of the study cohort

	**Mean ± SD, *n* (%)**
*n*	270
**Gender**	
female	57 (21.1%)
male	213 (78.9%)
**Age**	71.64 ± 9.71
**Surgery type**	
bilobectomy	4 (1.5%)
lobectomy	196 (72.6%)
pneumonectomy	29 (10.7%)
wedge resection	41 (15.2%)
**Chemotherapy**	
no	131 (48.5%)
yes	139 (51.5%)
**Radiotherapy**	
no	220 (81.5%)
yes	50 (18.5%)
**Histopathology**	
adenocarcinoma	118 (43.7%)
large cell carcinoma	30 (11.1%)
squamous cell carcinoma	122 (45.2%)
**Lymph node positivity**	
no	186 (68.9%)
yes	84 (31.1%)
**Clinical stage**	
IA	1 (0.4%)
IA1	10 (3.7%)
IA2	34 (12.6%)
IA3	35 (13.0%)
IB	19 (7.0%)
IIA	34 (12.6%)
IIB	64 (23.7%)
IIIA	44 (16.3%)
IIIB	7 (2.6%)
IVA	20 (7.4%)
IVB	2 (0.7%)
**Clinical stage group**	
advanced stage	73 (27.0%)
early stage	197 (73.0%)
**Vital status**	
alive	125 (46.3%)
dead	145 (53.7%)
**Time to death among deceased patients (months)**	32.63 ± 23.83
**1-year survival status**	
Alive	243 (90.0%)
Dead	27 (10.0%)
**2-year survival status**	
alive	214 (79.3%)
dead	56 (20.7%)
**5-year survival status**	
alive	144 (53.3%)
dead	126 (46.7%)
**PLR**	169.50 ± 84.43
**PAR**	79.47 ± 30.68
**NLR**	3.62 ± 2.66
**CAR**	5.50 ± 7.84
**mGPS**	
0	154 (57.0%)
1	88 (32.6%)
2	28 (10.4%)

**Abbreviations:** CAR, C-reactive protein-to-albumin ratio; mGPS, modified Glasgow Prognostic Score; n, number; NLR, neutrophil-to-lymphocyte ratio; PAR, platelet-to-albumin ratio; PLR, platelet-to-lymphocyte ratio; SD, standard deviation.

### Statistical analysis

All statistical analyses were performed using Wistats v3.0 (WisdomEra Corp., Istanbul, Turkey), a Python-based analytical platform integrating SciPy, scikit-learn, and statsmodels libraries (https://wistats.wisdomera.io, accessed on March 10, 2026). Distributional properties of continuous variables were assessed using skewness, kurtosis, and the Shapiro–Wilk test.

Categorical variables are presented as frequencies and percentages. Continuous variables are expressed as mean and standard deviation for normally distributed data, and as median with interquartile range for non-normally distributed data.

Comparisons between groups based on 1-year, 2-year, and 5-year survival status were performed using the chi-square test or Fisher’s exact test for categorical variables, and the independent samples *t*-test or Mann–Whitney *U* test for continuous variables, as appropriate. The predefined survival endpoints were selected to represent short-term (1-year), intermediate-term (2-year), and long-term (5-year) postoperative outcomes in surgically treated NSCLC.

To identify factors associated with time-specific survival at 1, 2, and 5 years, univariate and multivariable logistic regression analyses were performed. In these analyses, survival status was used as the dependent variable, with “alive” coded as 1 and “dead” as 0. Accordingly, odds ratios (ORs) greater than 1 indicate an increased likelihood of survival, while ORs less than 1 indicate a reduced likelihood of survival and thus a higher mortality risk. Odds ratios, therefore, represent the likelihood of survival, with values below 1 signifying reduced survival probability (i.e., increased mortality risk). Variables demonstrating statistical significance in univariate analysis were entered into multivariable models. Odds ratios with 95% confidence intervals were reported. All tests were two-tailed, and a *P*-value below 0.05 was considered statistically significant.

The primary objective of the regression analyses was to identify factors associated with predefined survival status at 1, 2, and 5 years following surgery. Based on prior literature and biological plausibility, CAR and mGPS were considered the primary inflammation-based biomarkers of interest, whereas analyses involving NLR, PLR, and PAR were considered exploratory and hypothesis-generating. Therefore, logistic regression models were applied using these fixed-time survival endpoints, which were intended to evaluate time-specific survival status rather than the exact timing of death. Overall survival patterns were additionally assessed using Kaplan–Meier survival analysis. Because survival status at the predefined 1-, 2-, and 5-year endpoints was fully ascertainable for all included patients, fixed-time logistic regression was considered appropriate for evaluating factors associated with these clinically relevant survival outcomes.

CAR and NLR were analyzed as continuous variables in all primary regression analyses. Median-based categorization was used exclusively for Kaplan–Meier visualization and descriptive survival comparisons. Overall survival was further evaluated using the Kaplan–Meier method to estimate survival probabilities over time. Survival curves were generated according to median-based categorization of CAR and NLR, as well as according to mGPS categories (0, 1, and 2). Differences between survival curves were assessed using the log-rank test. Survival time was defined as the interval between the date of surgery and death or last follow-up.

To visually evaluate time-dependent mortality patterns across inflammatory strata, mortality rates at 1, 2, and 5 years were calculated according to mGPS categories. Additionally, 5-year mortality rates were descriptively calculated across quartiles of CAR to illustrate mortality gradients across increasing CAR levels. Mortality proportions were expressed as percentages within each subgroup.

Multivariable models were constructed to evaluate the associations of candidate variables with the predefined survival endpoints. These analyses should be interpreted as exploratory assessments of associations with survival outcomes within the study cohort.

## Results

### Baseline characteristics of the study cohort

A total of 270 patients with surgically treated NSCLC were included in the final analysis (Table 1). The mean age was 71.64 ± 9.71 years, and the majority of patients were male (78.9%). Lobectomy was the most frequently performed surgical procedure (72.6%). Adenocarcinoma and squamous cell carcinoma were the predominant histopathological subtypes, accounting for 43.7% and 45.2% of cases, respectively. Lymph node positivity was observed in 31.1% of patients. Early-stage disease was present in 73.0% of the cohort, while 27.0% were classified as advanced stage.

During follow-up, 145 patients died, corresponding to an overall mortality rate of 53.7%. Among these 145 patients, the mean time from surgery to death was 32.63 ± 23.83 months. The survival rates at 1 year, 2 years, and 5 years were 90.0%, 79.3%, and 53.3%, respectively. Baseline inflammatory markers demonstrated mean values of 169.50 ± 84.43 for PLR, 79.47 ± 30.68 for PAR, 3.62 ± 2.66 for NLR, and 5.50 ± 7.84 for CAR. According to mGPS classification, 57.0% of patients were categorized as score 0, 32.6% as score 1, and 10.4% as score 2.

### Overall survival analysis according to inflammatory indices

Kaplan–Meier survival analysis demonstrated a clear separation of survival curves according to preoperative inflammatory markers (Figure 2). Patients with high CAR values exhibited significantly reduced overall survival compared with those in the low CAR group. A similar survival disadvantage was observed in patients with elevated NLR. When stratified by mGPS categories, a stepwise decline in survival probability was evident across increasing mGPS scores, with the poorest outcomes observed in patients with mGPS 2. These findings visually confirm the graded association between systemic inflammatory burden and overall survival in surgically treated NSCLC.

**Figure 2.**
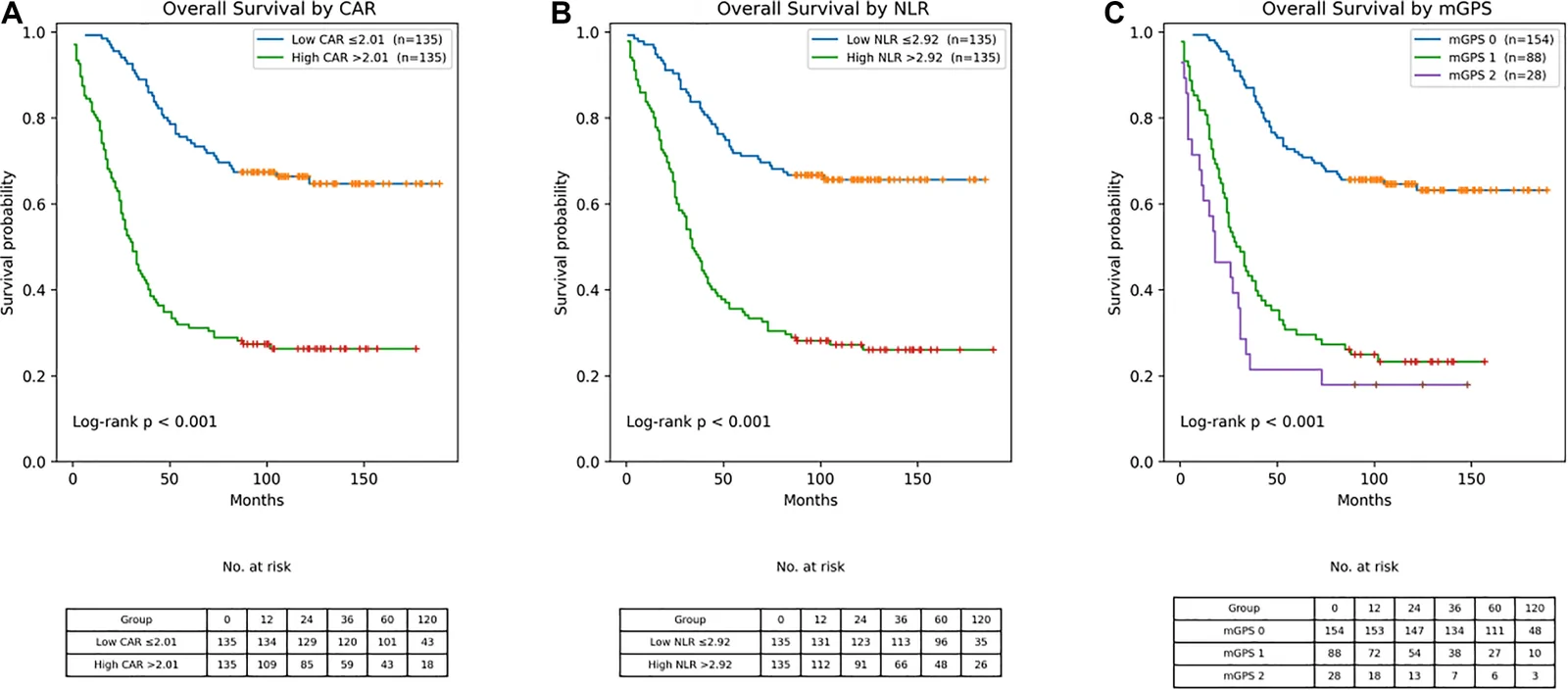
**Kaplan–Meier overall survival curves stratified by preoperative inflammation-based indices.** Overall survival was estimated from the date of surgical resection to death or last follow-up in 270 patients with non–small cell lung cancer. (A) Patients with high CAR (>2.01; *n* ═ 135) showed significantly lower overall survival than those with low CAR (≤2.01; *n* ═ 135; log-rank *P* < 0.001). (B) Patients with high NLR (>2.92; *n* ═ 135) showed significantly lower overall survival than those with low NLR (≤2.92; *n* ═ 135; log-rank *P* < 0.001). (C) Overall survival declined progressively across increasing mGPS categories (mGPS 0, *n* ═ 154; mGPS 1, *n* ═ 88; mGPS 2, *n* ═ 28; log-rank *P* < 0.001), with the poorest survival observed in patients with mGPS 2. CAR and NLR were dichotomized using median values for Kaplan–Meier visualization. Censoring marks are shown on the survival curves, and numbers at risk are presented below each panel. **Abbreviations:** CAR, C-reactive protein-to-albumin ratio; mGPS, modified Glasgow Prognostic Score; n, number; NLR, neutrophil-to-lymphocyte ratio.

### Comparative outcomes by one-year survival

At one year, 27 patients (Table 2) had died. Mortality at this juncture was significantly associated with male sex and advanced clinical stage. Patients who succumbed within the first year exhibited substantially elevated inflammatory indices compared to survivors. Mean PLR, NLR, and CAR values were significantly higher in the deceased cohort, and the distribution of mGPS categories differed markedly between groups. A pronounced concentration of deaths was observed among patients with mGPS scores of 1 and 2, whereas the majority of survivors were classified as mGPS 0.

**Table 2:** Comparative outcomes between living and deceased groups based on one-year survival rates

	**alive** **Mean ± SD, *n* (%)**	**dead** **Mean ± SD, *n* (%)**	***P***
**n**	243	27	
**Gender**			**0.047**
female	55 (22.6%)	2 (7.4%)	
male	188 (77.4%)	25 (92.6%)	
**Age**	71.28 ± 9.74	74.93 ± 8.97	0.089
**Surgery type**			0.742
bilobectomy	4 (1.6%)	0 (0.0%)	
lobectomy	176 (72.4%)	20 (74.1%)	
pneumonectomy	25 (10.3%)	4 (14.8%)	
wedge	38 (15.6%)	3 (11.1%)	
**Chemotherapy**			0.084
no	114 (46.9%)	17 (63.0%)	
yes	129 (53.1%)	10 (37.0%)	
**Radiotherapy**			0.222
no	196 (80.7%)	24 (88.9%)	
yes	47 (19.3%)	3 (11.1%)	
**Histopathology**			0.741
adenocarcinoma	108 (44.4%)	10 (37.0%)	
large cell carcinoma	27 (11.1%)	3 (11.1%)	
squamous cell carcinoma	108 (44.4%)	14 (51.9%)	
**Clinical stage group**			**0.009**
advanced stage	60 (24.7%)	13 (48.1%)	
early stage	183 (75.3%)	14 (51.9%)	
**PLR**	163.59 ± 81.49	222.71 ± 93.18	**0.001**
**PAR**	78.03 ± 28.77	92.38 ± 42.95	0.073
**NLR**	3.35 ± 2.33	6.02 ± 4.02	**<0.001**
**CAR**	4.37 ± 6.42	15.69 ± 11.51	**<0.001**
**mGPS**			**<0.001**
0	153 (63.0%)	1 (3.7%)	
1	72 (29.6%)	16 (59.3%)	
2	18 (7.4%)	10 (37.0%)	

**Abbreviations:** CAR, C-reactive protein-to-albumin ratio; mGPS, modified Glasgow Prognostic Score; n, number; NLR, neutrophil-to-lymphocyte ratio; PAR, platelet-to-albumin ratio; PLR, platelet-to-lymphocyte ratio; SD, standard deviation.

### Comparative outcomes by two-year survival

At two years, 56 patients (Table 3) had died. Male sex, advanced clinical stage, and histopathological subtype were significantly associated with mortality. Inflammatory markers remained consistently higher in patients who died. Significant differences were observed for PLR, PAR, NLR, and CAR. The mGPS demonstrated a strong graded association with two-year mortality, with progressively higher mortality rates across increasing mGPS categories (Table 3).

**Table 3:** Comparative outcomes between survivors and non-survivors at two-year follow-up

	**alive** **Mean ± SD, *n* (%)**	**dead** **Mean ± SD, *n* (%)**	***P***
***n***	214	56	
**Gender**			**0.021**
female	51 (23.8%)	6 (10.7%)	
male	163 (76.2%)	50 (89.3%)	
**Age**	71.04 ± 9.97	73.93 ± 8.35	0.072
**Surgery type**			0.489
bilobectomy	4 (1.9%)	0 (0.0%)	
lobectomy	158 (73.8%)	38 (67.9%)	
pneumonectomy	21 (9.8%)	8 (14.3%)	
wedge	31 (14.5%)	10 (17.9%)	
**Chemotherapy**			0.135
no	108 (50.5%)	23 (41.1%)	
yes	106 (49.5%)	33 (58.9%)	
**Radiotherapy**			0.203
no	177 (82.7%)	43 (76.8%)	
yes	37 (17.3%)	13 (23.2%)	
**Histopathology**			**0.002**
adenocarcinoma	101 (47.2%)	17 (30.4%)	
large cell carcinoma	17 (7.9%)	13 (23.2%)	
squamous cell carcinoma	96 (44.9%)	26 (46.4%)	
**Clinical stage group**			**0.037**
advanced stage	52 (24.3%)	21 (37.5%)	
early stage	162 (75.7%)	35 (62.5%)	
**PLR**	162.69 ± 83.93	195.54 ± 81.94	**0.003**
**PAR**	77.47 ± 28.99	87.10 ± 35.70	**0.032**
**NLR**	3.21 ± 2.12	5.20 ± 3.74	**<0.001**
**CAR**	3.54 ± 5.35	12.98 ± 10.86	**<0.001**
**mGPS**			**<0.001**
0	147 (68.7%)	7 (12.5%)	
1	54 (25.2%)	34 (60.7%)	
2	13 (6.1%)	15 (26.8%)	

**Abbreviations:** CAR, C-reactive protein-to-albumin ratio; mGPS, modified Glasgow Prognostic Score; n, number; NLR, neutrophil-to-lymphocyte ratio; PAR, platelet-to-albumin ratio; PLR, platelet-to-lymphocyte ratio; SD, standard deviation.

### Comparative outcomes by five-year survival

At five years, 126 patients (Table 4) had died. Advanced clinical stage was strongly associated with mortality. Chemotherapy and radiotherapy were more frequently observed among patients who died at five years, likely reflecting more aggressive or advanced disease characteristics. Inflammatory markers continued to show significant associations with mortality. PLR, NLR, and CAR values were significantly higher among non-survivors. The mGPS remained significantly associated with long-term mortality, and a clear stepwise increase in death rates was observed with increasing mGPS score.

**Table 4:** Comparative outcomes between surviving and deceased groups: A 5-year survival analysis

	**alive** **Mean ± SD, *n* (%)**	**dead** **Mean ± SD, *n* (%)**	***P***
*n*	144	126	
**Gender**			**0.033**
female	37 (25.7%)	20 (15.9%)	
male	107 (74.3%)	106 (84.1%)	
**Age**	71.19 ± 10.01	72.15 ± 9.38	0.620
**Surgery type**			0.983
bilobectomy	2 (1.4%)	2 (1.6%)	
lobectomy	106 (73.6%)	90 (71.4%)	
pneumonectomy	15 (10.4%)	14 (11.1%)	
wedge	21 (14.6%)	20 (15.9%)	
**Chemotherapy**			**0.002**
no	82 (56.9%)	49 (38.9%)	
yes	62 (43.1%)	77 (61.1%)	
**Radiotherapy**			**0.002**
no	127 (88.2%)	93 (73.8%)	
yes	17 (11.8%)	33 (26.2%)	
**Histopathology**			**0.038**
adenocarcinoma	70 (48.6%)	48 (38.1%)	
large cell carcinoma	10 (6.9%)	20 (15.9%)	
squamous cell carcinoma	64 (44.4%)	58 (46.0%)	
**Clinical stage group**			**<0.001**
advanced stage	27 (18.8%)	46 (36.5%)	
early stage	117 (81.2%)	80 (63.5%)	
**PLR**	154.95 ± 74.02	186.13 ± 92.47	**0.003**
**PAR**	75.61 ± 25.97	83.87 ± 34.90	0.074
**NLR**	2.91 ± 2.07	4.43 ± 3.01	**<0.001**
**CAR**	3.08 ± 5.15	8.27 ± 9.36	**<0.001**
**mGPS**			**<0.001**
0	111 (77.1%)	43 (34.1%)	
1	27 (18.8%)	61 (48.4%)	
2	6 (4.2%)	22 (17.5%)	

**Abbreviations:** CAR, C-reactive protein-to-albumin ratio; mGPS, modified Glasgow Prognostic Score; n, number; NLR, neutrophil-to-lymphocyte ratio; PAR, platelet-to-albumin ratio; PLR, platelet-to-lymphocyte ratio; SD, standard deviation.

### Regression analyses for time-specific survival

In univariate analysis for one-year survival, elevated CAR, NLR, PLR, PAR, and advanced clinical stage were significantly associated with reduced odds of survival (Table 5). In the multivariable model, both CAR and high mGPS status were independently associated with reduced odds of one-year survival.

**Table 5:** Univariate and multivariable logistic regression analyses of factors associated with 1-year, 2-year, and 5-year survival

	**Variable**	**OR**	**95% CI**	***P* value**
	PAR	0.988	0.977–0.998	**0.025**
	mGPS Status	0.02	0.00–0.17	**<0.001**
	Clinical stage group	0.35	0.16–0.79	**0.012**
	Gender	3.66	0.84–15.93	0.084
**1-year univariate**	CAR	0.88	0.84–0.93	**<0.001**
	Radiother apy	1.92	0.55–6.64	0.304
	NLR	0.79	0.71–0.89	**<0.001**
	PLR	0.994	0.990–0.997	**0.001**
	Chemother apy	1.92	0.85–4.37	0.118
	Age	0.96	0.92–1.00	0.065
	CAR	0.94	0.90–0.99	**0.024**
	Clinical stage group	0.54	0.21–1.39	0.202
**1-year multivariate**	NLR	0.88	0.75–1.03	0.112
	PAR	0.990	0.980–1.020	0.759
	PLR	0.998	0.994–1.013	0.163
	mGPS status	0.04	0.01–0.40	**0.005**
	**Variable**	**OR**	**95% CI**	***P*-value**
	PAR	0.991	0.982–1.000	**0.040**
	mGPS status	0.07	0.03–0.15	**<0.001**
	Clinical stage group	0.53	0.29–1.00	**0.050**
	Gender	2.61	1.06–6.43	0.038
**2-year univariate**	CAR	0.85	0.81–0.89	**<0.001**
	Radiother apy	0.69	0.34–1.41	0.311
	NLR	0.78	0.70–0.88	**<0.001**
	PLR	0.996	0.993–0.999	**0.012**
	Chemother apy	0.68	0.38–1.24	0.212
	Age	0.97	0.94–1.00	**0.049**
	Age	0.98	0.94–1.02	0.419
	CAR	0.93	0.88–0.98	**0.011**
	Clinical stage group	0.91	0.42–2.00	0.821
	Gender	1.73	0.59–5.01	0.316
**2-year multivariate**	NLR	0.87	0.74–1.02	**0.078**
	PAR	0.993	0.981–1.010	0.934
	PLR	0.996	0.990–1.011	0.786
	mGPS status	0.15	0.05–0.40	**<0.001**
	**Variable**	**OR**	**95% CI**	***P*-value**
	PAR	0.991	0.983–0.999	**0.030**
	mGPS Status	0.15	0.09–0.26	**<0.001**
**5-year univariate**	Clinical stage group	0.40	0.23–0.70	**0.001**
	Gender	1.83	1.00–3.36	**0.050**
	CAR	0.88	0.83–0.92	**<0.001**
	Radiother apy	0.38	0.20–0.72	**0.003**
	NLR	0.72	0.62–0.84	**<0.001**
	PLR	0.995	0.992–0.998	**0.003**
	Chemother apy	0.48	0.30–0.78	**0.003**
	Age	0.99	0.97–1.01	0.419
	CAR	0.98	0.93–1.03	0.437
	Chemother apy	1.93	1.02–3.65	**0.044**
	Clinical stage group	0.70	0.36–1.37	0.302
	NLR	0.80	0.68–0.95	**0.013**
**5-year multivariate**	PAR	0.992	0.980–1.012	0.410
	PLR	0.996	0.990–1.020	0.976
	Radiother apy	1.81	0.81–4.06	0.147
	mGPS status	0.23	0.11–0.47	**<0.001**

Odds ratios for binary variables were calculated relative to predefined reference categories: Male sex for gender, early-stage disease for clinical stage group, no chemotherapy for chemotherapy status, and no radiotherapy for radiotherapy status. Odds ratios exceeding 1 suggest increased survival odds, while values below 1 indicate a reduced probability of survival. mGPS status was modeled as a binary variable, with a low mGPS (score 0) serving as the reference category and a high mGPS (scores 1–2) coded as the exposed group. For continuous variables, odds ratios represent the estimated change in survival odds associated with a one-unit increase in the corresponding biomarker. **Abbreviations:** CAR, C-reactive protein-to-albumin ratio; CI, confidence interval; mGPS, modified Glasgow Prognostic Score; NLR, neutrophil-to-lymphocyte ratio; OR, odds ratio; PAR, platelet-to-albumin ratio; PLR, platelet-to-lymphocyte ratio.

For two-year survival, univariate analysis demonstrated significant associations between survival status and age, male sex, PAR, NLR, PLR, and CAR, while clinical stage showed borderline significance (*P* ═ 0.050). In multivariable analysis, CAR and high mGPS status remained independently associated with reduced two-year survival, whereas NLR showed borderline significance.

In the five-year analysis, univariate models demonstrated significant associations for mGPS, advanced clinical stage, chemotherapy, radiotherapy, PAR, PLR, NLR, and CAR, while sex showed borderline significance. After multivariable adjustment, high mGPS status and NLR remained independently associated with reduced long-term survival, whereas CAR was no longer independently associated with five-year survival.

### Time-specific mortality patterns

Time-specific mortality patterns according to inflammation-based indices are illustrated in Figure 3. Mortality rates increased progressively across mGPS categories at one, two, and five years, demonstrating a consistent gradient effect over time. Additionally, five-year mortality was descriptively higher across increasing CAR quartiles, with the highest mortality observed in the upper quartile.

**Figure 3.**
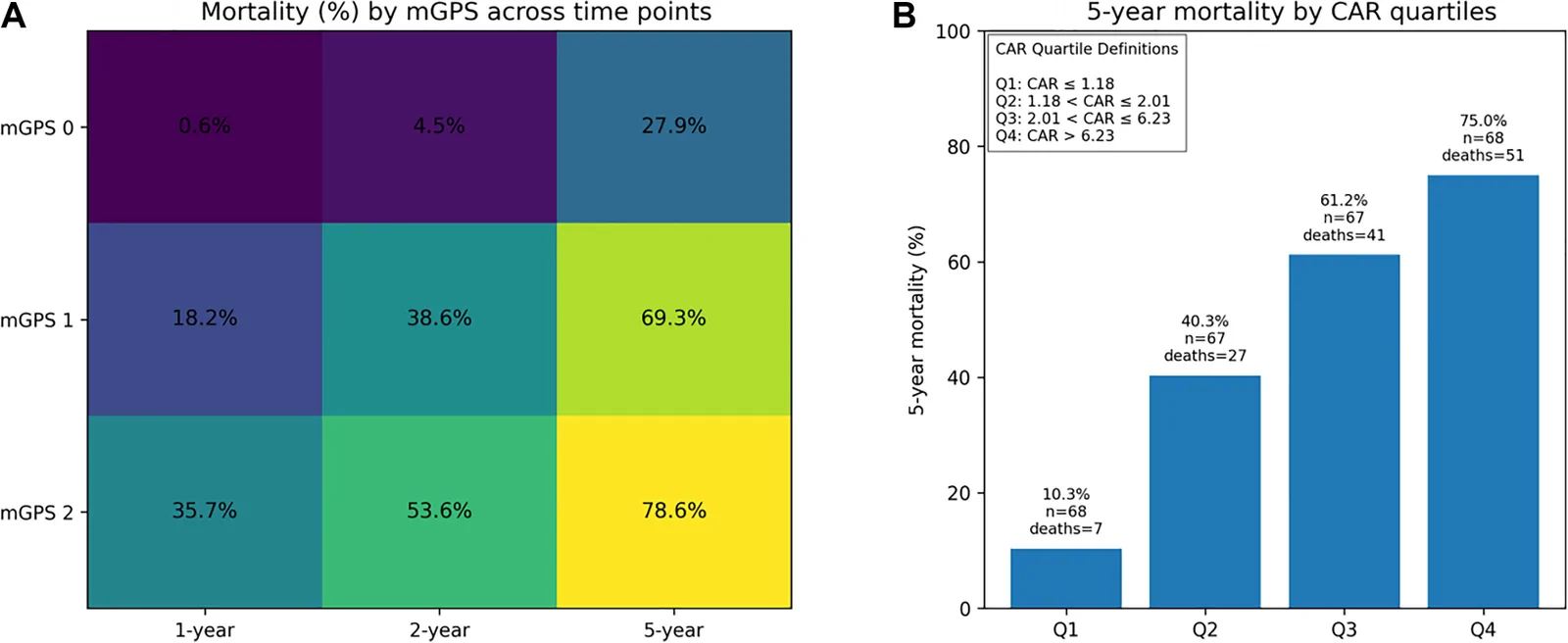
**Time-specific mortality according to preoperative inflammation-based indices.** (A) Heatmap showing mortality proportions at 1, 2, and 5 years after surgical resection in patients with NSCLC, stratified by mGPS category. Mortality increased progressively with higher mGPS at each time point, indicating a graded relationship between systemic inflammatory burden and postoperative mortality. (B) Descriptive 5-year mortality rates stratified by CAR quartiles. Higher CAR quartiles were associated with stepwise higher 5-year mortality, with the highest mortality observed in Q4 (CAR >6.23). CAR quartile cutoffs are shown in the inset, and subgroup sample sizes (*n*) and numbers of deaths are annotated above each bar. **Abbreviations:** CAR, C-reactive protein-to-albumin ratio; mGPS, modified Glasgow Prognostic Score; n, number; NSCLC, non–small cell lung cancer; Q, quartile.

### Overall interpretation

Across all evaluated time points, systemic inflammatory markers consistently demonstrated an association with mortality in surgically treated NSCLC. Elevated mGPS status showed the most consistent independent association with survival across all evaluated time-specific models. CAR was independently associated with short- and intermediate-term survival, while NLR was independently associated with long-term survival. The mGPS also exhibited a graded relationship with survival in unadjusted analyses, further supporting the prognostic relevance of systemic inflammatory status in this patient population.

## Discussion

This study aimed to evaluate whether routinely available preoperative inflammation-based indices are associated with short-, intermediate-, and long-term survival following surgical resection for NSCLC. In a cohort of 270 surgically treated patients, with a mean age of 71.64 years and a predominantly male demographic (78.9%), overall mortality was 53.7%. Among deceased patients, the mean time from surgery to death was 32.63 months, with one-, two-, and five-year survival rates of 90.0%, 79.3%, and 53.3%, respectively. Across all evaluated time points, patients who died consistently exhibited higher systemic inflammatory indices. Elevated mGPS status demonstrated the most consistent association with survival after adjustment in the exploratory fixed-time logistic models, while CAR and NLR showed time-dependent associations.

A central finding of our study is the prognostic relevance of inflammation-based biomarkers across multiple time points, with elevated mGPS status showing the most consistent independent association with survival. In our cohort, CAR was significantly higher in patients who died at one, two, and five years. After multivariable adjustment, CAR remained associated with reduced survival at one and two years in the exploratory fixed-time logistic models, but not at five years. This aligns closely with the broader lung cancer literature, including a meta-analysis by Deng et al. demonstrating that elevated CAR is associated with worse overall survival and exhibits a particularly strong association in surgical subgroups [2]. Our results are also consistent with large operable NSCLC cohorts where preoperative CAR independently predicted both disease progression and death [3]. The prognostic role of CAR appears to persist even in older surgical populations, as demonstrated by Miyazaki et al. in patients aged >80 years, supporting its potential prognostic relevance in real-world surgical candidates [4]. Moreover, comparative work evaluating inflammation-based immunonutritional parameters has suggested that CAR may outperform GPS and mGPS in some resected NSCLC cohorts [12, 13]. Prognostic performance appears to vary according to study population, biomarker definitions, and statistical modeling strategies. In our study, elevated mGPS status demonstrated the most consistent independent association with survival across all evaluated time-specific models, whereas CAR remained independently associated with short- and intermediate-term survival.

Our findings also support the prognostic relevance of NLR, particularly beyond the first year post-surgery. In our study, NLR was significantly higher in deceased patients across all time points. After multivariable adjustment, NLR showed borderline significance at two years and remained associated with reduced survival at five years. This is consistent with the seminal surgical cohort reported by Sarraf et al., where increasing preoperative NLR remained an independent predictor of survival following complete resection [6]. The prognostic utility of NLR in operable disease has also been validated in studies incorporating NLR alongside standard clinicopathological factors, demonstrating that systemic inflammation may capture prognostic information not fully reflected by stage alone [7]. Large institutional cohorts further support the association of pretreatment NLR with reduced survival in stage I–III NSCLC treated surgically [14]. More recent multi-institutional work indicates that NLR not only correlates with survival but that its postoperative evolution may also impact outcomes, suggesting that dynamic immune-inflammatory patterns may carry additional prognostic value beyond a single baseline measurement [15–17]. In high-risk surgical subgroups, such as pneumonectomy or sleeve lobectomy, elevated NLR has also been linked to poor prognosis, supporting the plausibility that stronger surgical stress and tumor burden may accentuate the clinical impact of systemic inflammation [18].

Platelet-related inflammatory indices in our cohort showed consistent unadjusted differences between alive and deceased groups, with PLR being higher among non-survivors at one, two, and five years, and PAR demonstrating significant differences at intermediate and long-term time points. These findings are directionally concordant with meta-analysis evidence suggesting that elevated PLR is associated with shorter overall survival in NSCLC [8]. Regarding PAR, prior work has proposed PAR as an independent prognostic biomarker in surgically treated NSCLC [19]. Recent studies have further expanded the concept of combined immune-inflammatory-nutritional assessment in surgically treated NSCLC, demonstrating prognostic value for composite indices that integrate systemic inflammation and nutritional status [20, 21]. In our multivariable analyses, however, platelet-based indices did not retain independent significance when modeled alongside CAR, NLR, and mGPS status. This may indicate collinearity among inflammation-based markers or that these markers better capture the inflammatory and nutritional components most relevant to mortality risk in this cohort. This pattern is also compatible with the notion that composite indices integrating CRP and albumin may provide a more stable signal than ratios derived solely from blood cell counts, which can be influenced by perioperative factors, transient infections, or unmeasured comorbid inflammatory conditions.

To facilitate interpretation in regression analyses, mGPS was dichotomized into low (0) vs high (1–2) status. Elevated mGPS was significantly associated with reduced survival, consistent with descriptive analyses and survival curves. Unadjusted analyses revealed a clear graded association between mGPS and survival, with higher mortality concentrated among patients with mGPS 1–2, supporting the prognostic relevance of systemic inflammatory status. While Kaplan–Meier curves suggested poorer survival for mGPS 2 compared to mGPS 1, the limited number of patients in the highest mGPS category precluded reliable evaluation of differences between these subgroups. This aligns with prior evidence linking GPS to overall survival in resectable NSCLC [10] and meta-analytic findings supporting mGPS’s prognostic value across lung cancer populations [11]. In this study, elevated mGPS status remained an independent predictor of survival across all evaluated time-specific models, suggesting that the combined inflammatory and nutritional information captured by mGPS offers robust prognostic insights beyond individual laboratory markers.

Our survival estimates provide clinically interpretable benchmarks for a surgical NSCLC cohort with a relatively older age distribution. The observed 5-year survival rate of 53.3% is broadly compatible with the expected prognosis in mixed-stage surgically treated NSCLC populations. The time-dependent separation by inflammatory indices suggests that systemic inflammation may contribute to both early and late mortality risk. The expected association between advanced stage and mortality across time points in our study is consistent with prior operable NSCLC literature, where stage was an important unadjusted determinant of survival but did not retain independent significance in multivariable models. Importantly, our findings indicate that inflammation markers provide prognostic information beyond disease stage. Elevated mGPS status remained independently associated with survival across all evaluated time-specific models, while CAR and NLR demonstrated time-dependent independent associations. Any observed associations involving chemotherapy or radiotherapy should be interpreted cautiously, as treatment allocation was non-random and influenced by clinical indications, disease stage, pathological risk factors, and multidisciplinary decisions. Therefore, these findings should not be interpreted as causal treatment effects but rather as associations reflecting underlying disease severity and treatment selection practices. Direct comparison of effect sizes across studies requires caution due to substantial variations in patient selection, stage distribution, surgical procedures, biomarker cut-off definitions, follow-up duration, and statistical modeling approaches in previous investigations.

Several limitations warrant consideration. First, this retrospective single-center study limits generalizability and introduces potential residual confounding despite multivariable adjustment. Second, inflammation-based indices can be influenced by intercurrent infection, chronic inflammatory disease, medication use, and perioperative physiological stress, factors not always fully captured in routine records. Third, we utilized laboratory values recorded closest to surgical admission; however, temporal variability in CRP, albumin, and leukocyte subpopulations may affect risk estimation. Dynamic changes in these markers, as suggested by studies examining postoperative inflammatory trajectories [15], might offer additional prognostic value. Fourth, while postoperative CRP has been explored as a prognostic biomarker in resected NSCLC, the literature is heterogeneous regarding timing, thresholds, and directionality of effect, and we did not incorporate postoperative CRP measurements into our models [22–24]. Additionally, the study database lacked sufficiently standardized information regarding resection margin status, extent of lymph node dissection, or detailed indications for surgery among all advanced-stage patients. Separate analyses excluding stage IV disease were not performed. Consequently, findings reflect the overall surgical cohort rather than specific pathological or treatment-defined subgroups. Minor variability in the timing of preoperative blood sampling may have occurred due to the use of routine clinical records. Information on biomarker collection timing relative to potential neoadjuvant treatment was not uniformly available in the retrospective records. Similarly, chemotherapy and radiotherapy variables could not be consistently classified by treatment timing. Therefore, the possible influence of preoperative systemic therapy on inflammatory biomarker values cannot be entirely excluded. However, all biomarker values were derived from preoperative assessments performed before surgical treatment and selected according to a predefined extraction protocol. Furthermore, several inflammation-based indices evaluated in this study share related biological pathways and partially overlapping laboratory components, suggesting some degree of correlation between them. The relatively small number of deaths observed in the 1-year analysis should be considered when interpreting multivariable model estimates, as model stability may be limited for this endpoint. Regression analyses were designed to evaluate survival status at predefined clinical time points rather than the continuous time-to-event process. Thus, reported odds ratios should be interpreted as measures of association with fixed-time survival status, not as hazard estimates from Cox proportional hazards models. The complete-case analytical approach, which excluded patients with missing key exposure or outcome variables, means that selection bias related to missing data cannot be entirely excluded. As this study was an exploratory evaluation of inflammation-based prognostic indices, regression analyses were not based on a prespecified clinical adjustment set. Accordingly, findings involving secondary inflammatory indices should be interpreted cautiously and require independent validation. Therefore, observed associations are exploratory and require confirmation in larger cohorts using predefined multivariable modeling strategies incorporating established clinical prognostic factors. Although detailed clinical stage information was available, some analyses used broader stage groupings, potentially obscuring prognostic differences between individual TNM stage categories, which should be explored in future studies with larger stage-specific cohorts. Finally, other immunonutritional scores, such as the albumin–globulin score, have demonstrated prognostic relevance after pulmonary lobectomy but were not included in our primary analyses and could be evaluated in future work [25].

Future research should prioritize prospective, multi-center validation of inflammation-based risk stratification approaches incorporating mGPS status, CAR, and NLR in surgically treated NSCLC. Such studies should ideally include standardized timing of biomarker collection, structured comorbidity assessment, and disease recurrence outcomes. Integrating dynamic postoperative inflammatory measurements, treatment response variables, and molecular tumor features may clarify whether inflammatory markers reflect tumor biology, host susceptibility, or both. Comparative modeling approaches that directly evaluate the incremental value of these markers over established clinicopathological predictors may further support their clinical adoption in perioperative decision-making and follow-up intensity planning. Additional studies evaluating discrimination, calibration, external validation, and potential clinical utility are required before these biomarkers can be considered for routine prognostic decision support.

## Conclusion

In surgically treated NSCLC, systemic inflammation-based indices were consistently associated with mortality across 1-, 2-, and 5-year follow-up periods. Elevated mGPS status remained associated with survival across all evaluated time-specific models, while CAR was associated with short- and intermediate-term survival, and NLR was associated with long-term survival. These findings suggest that routinely available inflammation-based biomarkers may provide complementary prognostic information in resected NSCLC. However, prospective validation, external validation, and formal evaluation of clinical utility are required before implementation in routine clinical decision-making.

## Data Availability

Anonymized study data are available via the Istinye University Dataset Sharing Platform: https://dataset.istinye.edu.tr/dataset?did=86. Access is granted for research use under the platform’s licensing and data-sharing policies.
